# Development of a community health and wellness pilot in a subsidised seniors’ apartment building in Hamilton, Ontario: Community Health Awareness Program delivered by Emergency Medical Services (CHAP-EMS)

**DOI:** 10.1186/s13104-015-1061-8

**Published:** 2015-04-01

**Authors:** Gina Agarwal, Ricardo N Angeles, Beatrice McDonough, Brent McLeod, Francine Marzanek, Melissa Pirrie, Lisa Dolovich

**Affiliations:** Department of Family Medicine, Faculty of Health Sciences, McMaster University, McMaster Innovation Park, 175 Longwood Road South, Suite 201A, Hamilton, ON L8P 0A1 Canada; City of Hamilton, Public Health Services, Unit 8-1447 Upper Ottawa, Hamilton, ON L8W 3J6 Canada; Hamilton Paramedic Services, 1227 Stone Church East, Hamilton, Ontario L8W 2C6 Canada; Centre for Evaluation of Medicines, 105 Main Street East, Level P1, Hamilton, ON L8N 1G6 Canada

**Keywords:** Community paramedicine, Cardiovascular health, Diabetes risk, Falls risk, Health promotion, Lifestyle risk screening, Older adults, EMS calls, Health assessment, Seniors housing

## Abstract

**Background:**

Older adults have higher risk of developing cardiovascular disease, diabetes and falls, leading to costly emergency medical service (EMS) calls and emergency room visits. We developed the Community Health Assessment Program through EMS (CHAP-EMS) that focuses on health promotion/prevention of hypertension and diabetes, links with primary care practitioners, targets seniors living in subsidized housing, and aims to reduce morbidity from these conditions, thereby reducing EMS calls. In this pilot study, we evaluated the feasibility of implementing the CHAP-EMS, attendance rates, prevalence of high blood pressure and cardiovascular risk factors.

**Methods:**

In this pilot study the CHAP-EMS was implemented in the intervention site over a 12 month period. BP, lifestyle, cardiovascular risk and EMS call rates were collected and descriptive analyses performed. Participants were residents (low income seniors) of a subsidized housing complex in Hamilton, Ontario. Two paramedics provided once-weekly sessions, measuring BP, assessing diabetes/lifestyle risk (CANRISK questionnaire) and discussed prevention/local wellness activities in the intervention site. Follow up was invited.

**Results:**

A total of 1365 visits with 79 unique participants occurred; 48 (25.2%) visited at least twice; mean age was 72.2; 87.2% were 65 years of age and older and 68.1% were female; 90.3% had a family doctor. Overall, 45.2% had elevated BP initially from the total; 50.0% of participants previously diagnosed with hypertension had elevated BP while 33.3% not previously diagnosed had elevated BP. Almost 1 in 5 (19.4%) had diabetes; 66.7% had moderate to high risk of developing diabetes.

**Conclusion:**

This pilot study indicates that CHAP-EMS is a feasible program that could have impact on BP, lifestyle factors, diabetes risk and EMS calls in the buildings in which it was implemented.

## Background

Studies have shown that older adults account for more than a third of all Emergency Medical Services (EMS) calls [1-4]. Over 60% of reasons given for EMS calls have been related to cardiopulmonary conditions, diabetes, and falls related trauma [1-4]. Each EMS visit can cost up to $259 [5,6] and an additional $785 per ambulance trip to the hospital emergency rooms (ER) in Ontario [5]. Therefore, any interventions that reduce EMS calls and eventual ER visits could generate cost savings to the healthcare system.

Older adults living in subsidized housing units report poorer health from a multitude of chronic illnesses, such as CVD and diabetes, compared to those living in unsubsidized housing units [7]. Interacting psychosocial and physical factors, such as low income and advanced age, complicates utilization of community and healthcare services and are associated with higher risk of chronic illness and disability [8]. Older adults cannot easily find or access a primary care provider [9]. Team-based community paramedicine is one approach that supports primary care access for older adults through promoting areas of care that influence the health and well-being of older adults [10].

Studies regarding an expanded role of paramedics relating to health promotion have been limited to case studies and mostly conducted in rural areas in Australia [11-13]. In many cases, paramedics were seen as an underused human resource that could be employed in rural and remote areas [11]. Expanded roles included both primary care health promotion and management such as community education and engagement, preventive services, treatment of minor illness (for locally endemic conditions), and promotion of lifestyle change to prevent and manage chronic disease [11,13].

### The CHAP-EMS program

The Community Health Assessment Program through EMS (CHAP-EMS) was created to explore the feasibility of an expanded community paramedicine role within a high needs urban setting. CHAP-EMS is delivered by accommodated paramedics to older adult residents living in subsidized housing in Hamilton, Ontario, Canada.

CHAP-EMS is based on the Cardiovascular Health Awareness Program (CHAP), a blood pressure and cardiovascular screening program [14], and an extended version of this, the Community Health Awareness of Diabetes (CHAD) Program [15]. The Cardiovascular Health Awareness Program (CHAP) is a successful low cost health promotion program which targets cardiovascular risk factors, including the detection and management of hypertension. It combines individual- and population-level strategies for primary prevention and ‘closes the loop’ by linking participants to follow-up care. CHAP is a community-based, primary care-centered, volunteer peer-led, free of charge, CVD risk-assessment and blood pressure (BP) monitoring program combined with health education sessions for community dwelling older adults [14]. Literature demonstrates that the CHAP program resulted in significant 9% relative reduction in hospital admissions due to, heart failure, and heart attacks in people aged 65 and over [16]. The CHAD Program is an extended version of CHAP, including a diabetes risk assessment (the CANRISK tool) that is delivered using the same infrastructure. This component enabled 11% to be identified with diabetes [15].

The CHAP-EMS project expands the program to serve older adults living in subsidized housing, adding diabetes and falls screening, and utilizing accommodated paramedic personnel to run the program with the intent of lowering rates of EMS calls and ER visits. The paramedics running this program are ‘accommodated’ paramedics and unable to assume traditional paramedic duties due to personal limitations such as pregnancy or injuries. These limitations, though rendering traditional paramedic duties unsuitable, can still allow for simple health promotion work. Paramedics can accurately assess the patient’s health status and environmental context [17-21] to provide non-urgent health care services in areas of community need.

CHAP-EMS was developed through a series of consultative meetings with representatives of a core group of organizations responsible for health service delivery to older adults living in subsidized seniors’ buildings in Hamilton, Ontario. The organizations represented were Hamilton Paramedics, City Housing Hamilton, Hamilton Public Health Services, Community Care Access Centre (CCAC), and the Department of Family Medicine Research, McMaster University. This group served as the advisory committee for the program.

### Theoretical framework

A systematic review on integrated care for the elderly has highlighted the implementation of features of Wagner’s Chronic Care Model (CCM) [22]. The CCM is a primary–care based framework aimed at improving the care of persons with chronic illnesses. It integrates six elements (delivery system design, self-management support, decision support, clinical information services, community resources and health system organization) into a model designed to foster more productive interactions among health care providers and patients for more effective team care and improved health outcomes [23].

**Figure 1:**
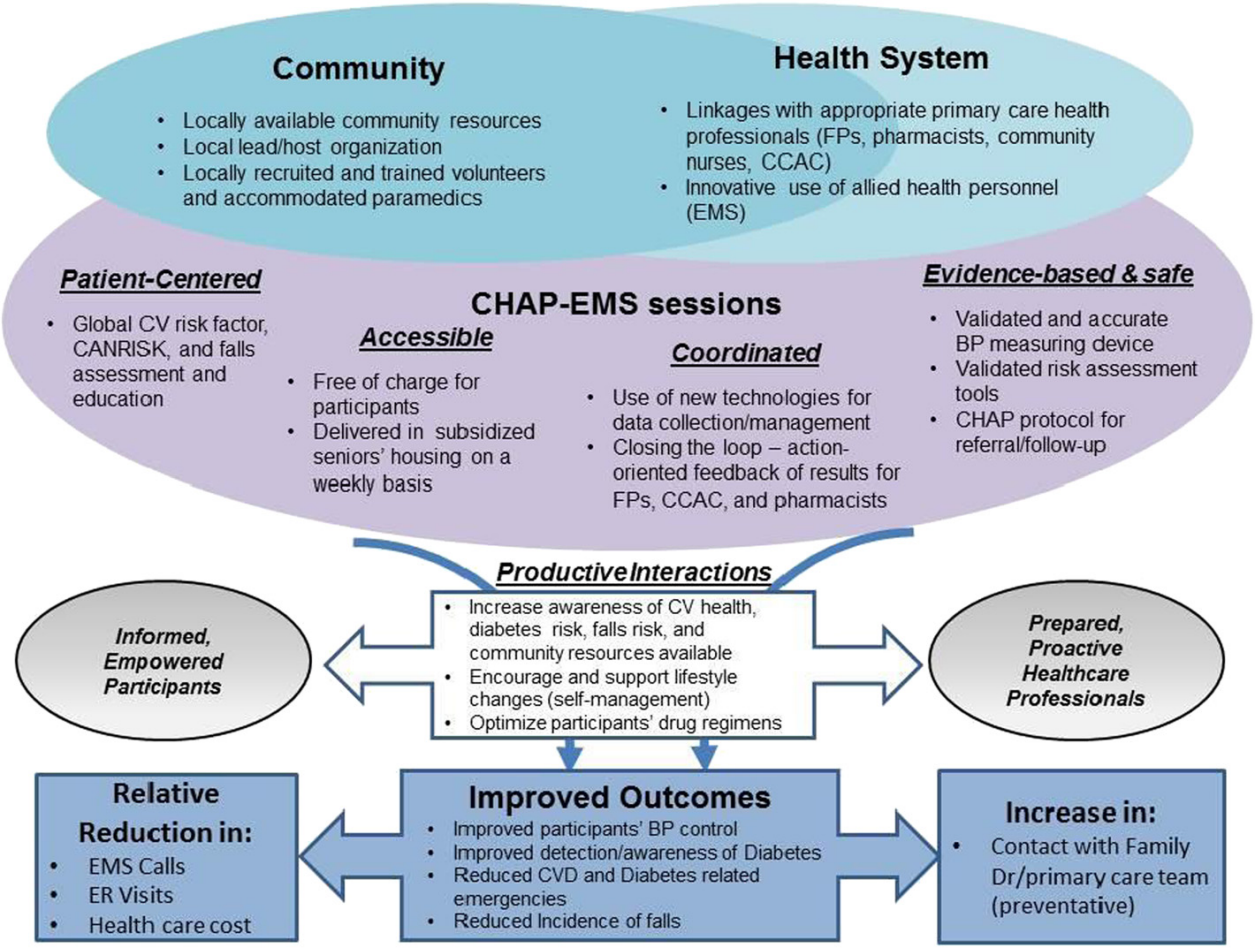
**Theoretical framework (uploaded as a separate file).**

This project has adopted the integrated CCM as a basis to explore the theorized impacts of CHAP-EMS on healthcare delivery that affect individuals and the greater system (see Figure 1). CHAP-EMS increases older adults’ awareness of their health risk factors, encourages them to manage these risk factors before any emergency event occurs, and links them with locally available community resources and services based on those risk factors. Furthermore the participants’ health information (with their consent) is sent to their primary care physician so that health care services (promotion, screening, and follow-up) are coordinated to improve participants’ health status. This is expected to bring better health outcomes leading to less adverse health issues, EMS calls, ER visits and hospital admissions.

## Methods

### Research design

This was a pilot study which measured the feasibility and challenges of implementing CHAP-EMS. This was done to provide information for the planning of a large scale randomized controlled trial to test the effectiveness of CHAP-EMS. Research ethics was granted by the Hamilton Integrated Research Ethics Board.

### Participants and setting

Participants were the residents of a subsidised seniors’ building in Hamilton (maximum capacity was approximately 250 residents), which was identified by City Housing Hamilton as having a high volume of EMS calls. Residents were invited to weekly program sessions by posters placed in the lobby, sitting room and by flyers delivered under their doors. There were no exclusion criteria for participation. People who were not able to speak English were encouraged to attend with a translator (family or neighbor). Flyers and posters were translated into Russian, Polish and Mandarin since these were 3 additional languages identified as being used by residents of the building. CHAP-EMS was provided free of charge for all building residents and was primarily targeted to people aged 65 years and older, and offered on an individual basis.

### Intervention

The main elements of CHAP-EMS include: 1) blood pressure, diabetes risk assessment; 2) falls risk assessment; 3) health education/promotion, goal setting and targeted referral to appropriate wellness sessions and community resources (e.g. CCAC); 4) identification and referral of patients at high risk; and 5) referral of participants’ health information to their regular physician. Two trained Hamilton accommodated paramedic professionals delivered the weekly CHAP-EMS sessions. The paramedic training included education to increase knowledge and skills regarding health promotion and disease prevention, assessment for cardiovascular, diabetes and falls risk, and awareness of local resources available to assist participants in addressing their identified risk factors. This half-day long training program was specifically developed for the CHAP-EMS program, by a family doctor and public health nurse in consultation with a paramedic.

The specific health-risk assessments included as part of the CHAP EMS intervention were blood pressure and cardiovascular risk screening, diabetes risk-assessment using the validated CANRISK tool [24], and the ‘Timed up and go’ (TUG) test [25,26]. Health assessments were conducted individually, in a semi-private setting behind a privacy screen in a common area. Information about each participant was collected after consent was obtained, and was collated in a database developed to calculate and summarize risk factors, to enable targeted health promotion education or community referrals to be delivered using a pre-specified algorithm. This algorithm was developed to direct participants to the appropriate services. Those identified as high risk (by virtue of high blood pressure, high diabetes risk score or high risk for falls from TUG assessment) during the CHAP-EMS session were immediately referred to timely and appropriate health care services for follow-up (such as their family physician, urgent care, community care access centre to gain immediate help for these pressing concerns). Participants with a moderate risk profile were referred to already existing community supports to assist them in managing their health, and included age-appropriate physical activity, healthy eating referrals, social engagement opportunities such as cooking demonstrations, community gardens and referrals to local community resources as required. Follow-up for identified concerns were provided through linkages with primary health care providers and CCAC community referrals. Geographical information system (GIS) mapping provided a visual portrait of local availability of health care resources and locations where older adults can be safely active and obtain healthy food choices.

Enrollment in the CHAP-EMS program was permitted at any time, and participation was encouraged by attendance more than once, at which times BP was re-checked, risk factors were re-addressed and CANRISK was re-assessed again at 6 months. All participants were encouraged to regularly attend CHAP-EMS program sessions for BP monitoring. Consenting participants had their BP and risk profile sent by secure fax from the CHAP-EMS database to their regular physicians. Those without regular physicians were referred to CCAC for assistance in registering with a suitable local family physician.

### Feasibility outcomes

Process measures were assessed. These included attendance rates to CHAP-EMS, characteristics of attendees, risk assessment results of participants, referrals to community resources, and challenges during implementation.

## Results

CHAP-EMS was implemented once-weekly for 1-year in the pilot study site. During the intervention period, there were a total of 1,365 participant visits to the intervention sessions by 79 unique participants (34.8%) out of 234 eligible participants (those who were over 65 years old and resident in the building). All who attended had consented. None consented and then did not attend. Table 1 summarizes the participant characteristics and results of the risk factor assessment.

**Table 1:** **Characteristics of participants attending CHAP-EMS sessions**

**Participant profile**	**N = 79**
Mean age (SD)	72.2 (12.1)
% Female	68.1
Education (%)	
• Some high school or less	33.3
• High school diploma	19.4
• Some college of more	29.2
• Not specified	17.1
% With family doctors	90.3
% Previously diagnosed with hypertension	58.3
% Previously diagnosed with diabetes	19.4

The mean age of the participants was 72.2; a large majority (87.2%) were 65 and older.

Most of the participants were female (68.1%) and 90.3% had a family doctor. The number of medications taken by the participants daily ranged from 0–12 medications.

Among the 79 participants, 19.4% already had diabetes while 66.7% had moderate to high risk of developing it based on the CANRISK assessment. Overall, 45.2% of the participants had elevated blood pressure (BP) during the initial visit; 50.0% of the participants who were previously diagnosed with hypertension had elevated BP while 33.3% of participants not previously diagnosed with hypertension had elevated BP. The prevalence of modifiable risk factors can be seen in Table 2.

**Table 2:** **Prevalence of modifiable risk factors among the participants**

**Risk factors**	**n (%)**
High waist circumference	46 (63.9)
Elevated body mass index	44 (61.1)
High level of stress	38 (52.8)
Elevated BP during initial visit	35 (45.2)
Low physical activity	30 (41.7)
High salt intake	25 (34.7)
High fat food intake	18 (33.3)
Low fruits & vegetable intake	16 (29.6)
Smoking	21 (29.2)

In terms of program feasibility, forty-eight (25.2%) had at least 2 or more visits to the program. On average, there were 3 to 5 new participants enrolled in the program every month. Participants who had specific risk factors were referred to available resources (exercise programs, food and nutrition resources, CCAC, social welfare organizations, public health, etc.). Most of the regular participants were referred to and enrolled in the in-house wellness program, run by a Local Seniors’ Program which included an exercise program and scheduled lectures. Participants noted to have elevated BP were recommended to see their family physicians. Three participants who were previously undiagnosed were provided with a diagnosis of hypertension and were subsequently treated with medication. Five participants previously diagnosed with hypertension had their medications adjusted. One participant was confirmed to have diabetes after the CANRISK assessment and 2 participants had their diabetes medications adjusted.

Thirty-four family physicians were contacted and asked if they wanted to participate with the CHAP-EMS program and receive their individual patient session-information collected through its database; 26 agreed to participate by receiving the information via fax.

## Discussion

The utilization of accommodated paramedics in the delivery of community based health promotion and management services using the CHAP model to vulnerable populations is feasible, novel, and appealing. Feasibility has been demonstrated by the fact that paramedics delivered the program for a year successfully, without staffing issues and adequate flow was sustained for participants throughout (the sessions were never empty). Furthermore, participants had potential cardiovascular risk factors that could benefit from health promotion.

In addition, the program was delivered one day a week by two accommodated paramedics (therefore at no extra cost to the service since these individuals were modified, and already being paid for being ‘off sick’ from regular paramedic duties), and the equipment required was only a laptop, an automated blood pressure machine, and capillary blood sugar testing materials; all of which are minor expenses. Indeed, due to the low cost nature of the program, we were able to conduct this as a pilot study without formal funding, only in kind contributions from the Department of Family Medicine and Hamilton Paramedicine service. Other programs centring on community paramedicine have used paramedics in a variety of situations, mainly around visiting patients in their homes after certain key health events. Our intervention incorporated usual health promotion activities; however the setting was quite different since we implemented our intervention in a fast-paced, densely populated urban setting. The partnership with the interdisciplinary collaborators was also novel and fundamental in identifying the setting and providing access to a vulnerable population most in need for the intervention. The development approach using collaborative partnerships is readily replicable in other communities.

There were a few challenges in implementing the program. One particular challenge was that the availability of accommodated/modified paramedic staff regularly changed requiring a flexible and easy to implement training program. However, though a cheaper solution, since extra salary support would not be required, accommodated paramedics are not the only paramedics to be able to deliver the program – of course, regular paramedics could also deliver it. Another solution to this availability concern currently being explored is adhering to the original CHAP program principle [14,16] of training peer health educators who would then deliver the program under the supervision or in partnership with a trained EMS staff. The benefits of this model have yet to be tried and tested. Another challenge involved the recruitment of residents to CHAP-EMS sessions. To further increase participation, research team members attended tenants’ association meetings and linked with housing staff to communicate with tenants about the program in their written and face to face interactions.

## Conclusion

In summary, CHAP-EMS was a feasible program, attracting a third of building residents, which is very reasonable and encouraging. Participants who required further follow-up for their health care were identified and also received links with community support services to address their cardiovascular risks. Using the existing resources of accommodated paramedics and the effective CHAP model, we have created an intervention in partnership with Hamilton Emergency Medical Services, City of Hamilton Housing and Public Health Services and the Community Care Access Center that focuses on *those particular* issues experienced by older adults that often lead to EMS calls. We suspect that this program could lead to a decrease in the number of EMS calls and ER visits in buildings with the program, having implications in terms of health care savings. We also believe that the program could improve the health behavior of building occupants which is expected to improve health outcomes. Such findings will have significant policy implications in favor of widespread implementation of this program. Potential limitations of this pilot study were the fact that feasibility can only be assessed in terms of this particular building and location and therefore may not be generalisable to other locations and situations. In addition, this implementation utilized accommodated paramedics for program delivery, which may not be feasible in other communities. Therefore we plan to conduct a randomized controlled trial in matched seniors housing building pairs to determine the efficacy of the program over one year, as well as the cost, compared to usual care. However, this program has the potential to deliver quality care and reduce the burden of chronic disease among the elderly living in subsidized housing.

## Abbreviations

BP: Blood pressure
CCAC: Community Care Access Centre
CCM: Chronic care model
CHAD: Community Health Awareness of Diabetes
CHAP: Cardiovascular Health Awareness Program
CHAP-EMS: Community Health Assessment through Emergency Medical Services
CVD: Cardiovascular
EMS: Emergency Medical Services
GIS: Geographical information system
TUG: Timed-up and go
